# Effect of Germinated Sorghum Extract on the Physical and Thermal Properties of Pre-Gelatinized Cereals, Sweet Potato and Beans Starches

**DOI:** 10.3390/molecules28207030

**Published:** 2023-10-11

**Authors:** Hesham Alqah, Shahzad Hussain, Mohamed Saleh Alamri, Abdellatif A. Mohamed, Akram A. Qasem, Mohamed A. Ibraheem, Aamir Shehzad

**Affiliations:** 1 Department of Food Science and Nutrition, King Saud University, Riyadh 11451, Saudi Arabia; heshamfrnd@gmail.com (H.A.); msalamri@ksu.edu.sa (M.S.A.); abdmohamed@ksu.edu.sa (A.A.M.); aqasem@ksu.edu.sa (A.A.Q.); mfadol@ksu.edu.sa (M.A.I.); 2 UniLaSalle, Univ. Artois, ULR7519—Transformations & Agro-Ressources, Normandie Université, F-76130 Mont-Saint-Aignan, France; aamir.shehzad@unilasalle.fr

**Keywords:** starch, enzyme, sorghum (*Sorghum bicolor* L.) extract, sorption

## Abstract

Starches from different botanical sources are affected in the presence of enzymes. This study investigated the impact of α-amylase on several properties of pre-gelatinized starches derived from chickpea (*Cicer arietinum* L.), wheat (*Triticum aestivum* L.), corn (*Zea mays* L.), white beans (*Phaseolus vulgaris*), and sweet potatoes (*Ipomoea batatas* L.). Specifically, the water holding capacity, freezable water content, sugar content, and water sorption isotherm (adsorption and desorption) properties were examined. The source of α-amylase utilized in this study was a germinated sorghum (*Sorghum bicolor* L. Moench) extract (GSE). The starch samples were subjected to annealing at temperatures of 40, 50, and 60 °C for durations of either 30 or 60 min prior to the process of gelatinization. A significant increase in the annealing temperature and GSE resulted in a notable enhancement in both the water-holding capacity and the sugar content of the starch. The ordering of starches in terms of their freezable water content is as follows: Chickpea starch (C.P.S) > white beans starch (W.B.S) > wheat starch (W.S) > chickpea starch (C.S) > sweet potato starch (S.P.S). The Guggenheim-Anderson-de Boer (GAB) model was only employed for fitting the data, as the Brunauer–Emmett–Teller (BET) model had a low root mean square error (RMSE). The application of annealing and GSE treatment resulted in a shift of the adsorption and desorption isotherms towards greater levels of moisture content. A strong hysteresis was found in the adsorption and desorption curves, notably within the water activity range of 0.6 to 0.8. The GSE treatment and longer annealing time had an impact on the monolayer water content (m_o_), as well as the C and K parameters of the GAB model, irrespective of the annealing temperature. These results can be used to evaluate the applicability of starch in the pharmaceutical and food sectors.

## 1. Introduction

Starch granules are normally insoluble in cold water, and thus cannot exhibit any of the main functions, such as increased viscosity and water binding at ambient temperature. These restrictions have limited the use of native starches in many items. Instant or pre-gelatinized starch (PGS) was used to overcome such issues which is often referred to as cold gel. This form of modified starch will swell in cold water which leads to a rapid viscosity increase depending on the solid content [1]. Pre-gelatinized starches are used in food processing for thickening or water retention without heat application. These starches are commonly used in puddings, and baby food preparation, especially cereal based designated for infants under 12 months of age [2,3]. The enzymatic hydrolysis promotes the partial degradation of starch in cereals prior to ingestion and facilitates starch digestion by infants due to the limited pancreas ability to digest starch [4]. Pre-gelatinization is accomplished by exposing a thin coating of starch slurry to a heated surface, such as a hot plate or drum dryer, with twin drum dryers producing a superior product than single drum dryers. The qualities of the finished product can be influenced by the slurry content, temperature, and speed of the drum [5,6]. The starch granules may be damaged or destroyed depending on the severity of the pre-gelatinization procedure. As a result, water easily interacts with the starch components, increasing viscosity without heating [1,7]. However, some limitations of PGS including grainy texture, inadequate consistency, and weak gels have restricted its applications to some foods. These deficiencies are mainly due to the disintegration of the granules and retrogradation of the wet starch film during drying [8].

The thermodynamic relationship between water activity and moisture balance of food at a constant temperature and pressure is represented by sorption isotherms since the quality of the stored products primarily relies on its water activity, which depends on its relative humidity and storage temperature. The moisture sorption isotherms of food products are useful information regarding their stability and prediction of their shelf life. At a given temperature, the isotherm provides information on the relation between humidity and the water activities [9,10]. Many models have been proposed to explain the moisture-sorption isotherm including multi-layered (Brunauer–Emmett–Teller (BET) and Guggenheim-Anderson-de Boer (GAB) models), semi-empirical (Ferro-Fontan, Henderson, and Halsey), or empirical (Smith and Oswin models). The BET isotherm model is the most important model for understanding the multi-layer sorption isotherm, in particular, for Type II isotherm [11]. The GAB model is known as the most flexible sorption model in the literature. The American Society of Agricultural Engineers has adopted the BET and GAB models for the description of sorption isotherms. These models are used extensively in the literature [12,13,14]. The study by Ocieczek, et al. [15] concluded that despite identical particle size characteristics, native cassava starch notably differs from potato starch in terms of hygroscopicity as indicated by the parameters of the BET model. The moisture sorption behavior of the pea starch films exhibited an upward trend as the water activity levels increased across various temperatures (5, 15, 25, and 40 °C), conforming to a Type III isotherm. The equilibrium moisture content and monolayer moisture contents (m_o_) exhibited a decrease as the storage temperature increased while maintaining a constant water activity [16].

This study aimed to evaluate the impact of crude germinated sorghum extract on the sorption isotherm, thermal characteristics, amount of freezable water, and water retention capacity of pre-gelatinized chickpea starches, corn, White beans, wheat, and sweet potato starches. The outcome of this study demonstrates that the cost of the procedure is significantly lower in comparison to utilizing a refined α-amylase extract.

## 2. Results and Discussion

### 2.1. Water Holding Capacity

Based on the activity of pure α-amylase solution, the concentration of α-amylase in the GSE was found to be 5 mg/10 mL. The percentage (%) of amylose content of the tested starches for wheat, chickpea, sweet potato, white beans, and corn, was 25.0, 24.0, 22.6, 20.9, and 20.4, respectively. The water holding capacity (WHC) is defined as the amount of water that can be absorbed per gram of sample [17]. The WHC of native, annealed, and GSE-treated pre-gelatinized (PGS) starches is presented in (Table 1). The range of the WHC was 13.0–4.51 (g/g) and 14.35–5.28 (g/g) for the native and annealed, respectively. Annealing appeared to increase WHC of chickpea starch, corn starch, and white bean starch by 12.9, 20.1, and 15.3%, respectively, but W.S and S.P.S exhibited a drop in WHC by 12.4 and 3.6%, respectively. The WHC of native and annealed starches rank as: S.P.S > C.P.S > W.B.S > W.S > C.S. According to these ranks, amylose content was not the determining factor in the WHC since high amylose content starch, such as wheat starch, did not exhibit highest WHC. Botanical origin could be considered a factor because wheat starch and corn starch, cereal-based starches, exhibited the lowest values, whereas sweet potato starch, and tuber starch, exhibited the highest values. The data presented here indicate that the amorphous region of wheat starch granules is more compact compared to sweet potato starch, and chickpea starch since it allowed limited water penetration. Starch granules bind water via hydrogen bonding. Therefore, the differences between the WHC can be attributed to the intensity of the hydrogen bonds and the accessibility of water to binding sites in the granule. Consequently, the WHC of starches is dependent on granule structure, botanical origin and type of processing (treatment), and to some extent amylose content. This is in agreement with previous reports which indicated that WHC, swelling power, and peak viscosity are correlated, but amylose content was not a major indicator of these parameters [18]. Alqah, et al. [19] reported that no correlation was found between WHC and peak viscosity of several starches. It is clear how annealing at 50 °C increased the WHC, compared to 40 and 60 °C which may indicate how this temperature affected the granule structure which could be attributed to an increase in granule porosity leading to higher WHC. The WHC has allegedly been higher in dry-heated starch compared to their native [20]. Overall, sweet potato starch behavior stood out because it was the most temperature sensitive, had the highest WHC, and ranked third with reference to amylose content. The effect of annealing on the WHC of chickpea starch was the most perceptible with or without GSE treatment compared to the other tested starches. All GSE-treated starches exhibited higher WHC, but GSE treatment appeared to have less impact on cereal-based starches (wheat and corn) (Table 1). Given that the α-amylase attack causes holes on the starch granule surface as well as the effect of higher temperatures, obviously increased the porosity of the granules and triggered higher WHC conditions which explains the increase. The ability of starch to bind and hold water is a desirable characteristic in the food industry especially when starch is used in frozen food products as stabilizers and emulsifiers because it prevents syneresis, therefore GSE treatment is a desirable process.

### 2.2. Sugars Content Determination

The sugar content (SC) of the native and treated starches is shown in (Table 2). Variation between the tested starches is clear, where annealed samples in GSE exhibited the highest sugar content due to the hydrolytic action of α-amylase. This variation indicates the level of susceptibility of the starch to α-amylase. Annealed samples exhibited SC lower than the native which indicated a loss of low molecular weight sugars during annealing at 60 °C for 60 min. The SC loss could be attributed to the swelling of the granules in the course of annealing which facilitates for leaching of lower molecular weight fractions. Annealing appeared to have a limited effect on the SC of chickpea starch, but GSE treatment resulted in the highest SC among the starches (Table 2). The SC rank of the native and annealed starches was: S.P.S > W.S > C.P.S > C.S > W.B.S, whereas GSE treated rank as: W.S > C.P.S > C.S > W.B.S > S.P.S. Based on this ranking, wheat starch was influenced the most with GSE by releasing the most sugar, whereas sweet potato starch released the minimum. Although native sweet potato starch contained the most SC, it was influenced more by annealing than GSE. Therefore, wheat starch was the most susceptible to GSE among the tested starches and sweet potato starch.

### 2.3. Freezable Water Determination

Modified PGS starch treated with GSE for 60 min at 40 °C was used for FW (freezable water) determination. One distinct DSC endotherm profile was obtained in all tested starches for FW. The size of the endotherm, onset, peak, and ΔH values shifted to higher or lower temperatures depending on the starch type (Table 3). These variations are reflective of the difference in the size of the peak of the melting ice. When sufficient amounts of water are present in the system appreciable amount of ice crystals form, but in limited water conditions most of the water is bound to starch with a small freezable quantity. This is dependent on the different water binding sites present in the pre-gelatinized starch (PGS) samples. These binding sites in PGS are typically hydroxyl groups and inter-glucose oxygen atoms. The interaction of these sites with water varies according to the molecular structure and compositional properties of the starches [21]. The freezable water contents (ΔH of melted ice/ΔH of water) of chickpea starch was the highest, whereas sweet potato starch exhibited the lowest value indicating more bound water (Table 3). The FW of the tested PGS rank as: C.P.S > W.B.S > W.S> C.S > S.P.S. When comparing the FW ranking to the starch amylose content, no connection was observed, which leads to the conclusion that granule structure is the main cause of the variation in the molecular structure of the PGS rather than the amylose content of the native starch. It is also true how physical modifications, such as gelatinization can be effective in separating starches based on water binding capacity. Obviously, gelatinization improves starch–water interaction. In addition, Fu, Wang, Zou, Li and Adhikari [21,22] reported that the gelatinization process breaks the weaker bonds in the amorphous region of the starch granule first, therefore increasing the hydration capacity of the starch. Researchers have observed that the completely gelatinized starch samples contain less freezable water (more bound water) than partially gelatinized samples. This is in agreement with literature reports [23]. Waxy starches exhibited less FW that common starch which indicates that amylopectin plays an important role in starch–water interaction, possibly due to the branching [24]. Although high amylose starches should exhibit high FW due to the low amylopectin, this was not true for W.S., because it has high amylose and relatively high FW. The high amylose–high FW theory is factual for chickpea starch. This could be attributed to the sensitivity of starch to the action of α–amylase. It is important to consider the action of GSE (α-amylase activity) and the sensitivity of the tested starches to α–amylase, because it changes the molecular structure of the PGS due to the enzymatic hydrolysis of native starch. The peak temperature of the melting ice showed variation, where PGS exhibited peak temperature higher than pure water except for W.S. This is consistent with the low FW of wheat starch.

### 2.4. Moisture Sorption Isotherms

**Figure 1:**
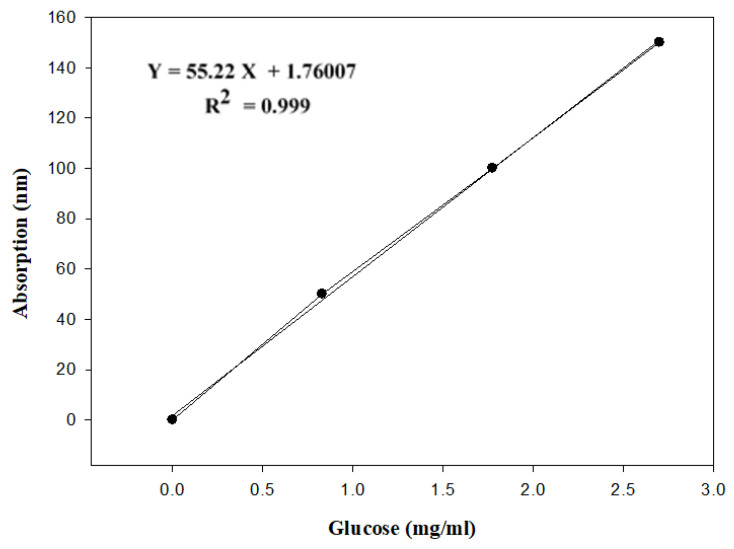
Glucose standard curve.

The desorption and adsorption isotherms profiles of the PGS demonstrate a simultaneous increase in equilibrium moisture content with increasing equilibrium relative humidity. This profile represents a sigmoidal shape, thus reflecting the dominant type II curve (Figure 1), according to the BET classification of isotherms [25]. The experimental quantitative evaluation of adsorption and desorption data were determined based on BET and GAB models, but the experimental data of BET of the present work exhibited a low coefficient of determination (R^2^); therefore, the current data represent the GAB model only. The experimental moisture isotherms data were fitted to GAB and BET models using nonlinear regression analysis. This theoretical tri-parametric model is suited for food engineering and is highly suitable for almost all foodstuffs with water activity ranging from 0.1 to 0.9 [26]. The parameters of the model also provide valuable details about the condition of water in food. For example, the definition of the monolayer moisture content (m_o_) is included in the GAB equation, which is linked to product stability and shelf life [27,28]. The data showed that annealing permitted greater enzyme accessibility to the amorphous and crystalline regions of starch granules, which promoted the idea of the development of a more porous structure, which in turn accelerated enzyme hydrolysis. It was, obvious how the equilibrium moisture content (EMC) of PGS tended to increase after treatment with 1.0 mL GSE for 60 min at all annealing temperatures. This could be attributed to the increase in the number of water binding sites induced by the enzyme (Table 4 and Table 5). The constant C is the total heat of sorption of the first layer of water vapor bound directly to the active binding sites, whereas K represents the multilayer water molecules with respect to the bulk water rather than vapor. The C value is always positive, and K is less than unity. The values of K and C presented here showed that GAB is suitable for fitting the pre-gelatinized starch data. The fit of GAB was evaluated by calculating the percentage square root error, RMSE, against experimental isothermal data, where GAB was found to be more fit than BET [29].

The chickpea starch isotherm data, presented in (Table 4), summarizes the estimated constants of the GAB model along with the root mean square error (RMSE) which indicates the absolute fit of the model. The coefficient of determination (R^2^) is also given in the table. The low values of RMSE or the R^2^ close to unity indicate that the GAB model is a good fit for the sorption isotherm data, and the projected parameters are statistically satisfactory. The monolayer moisture content (m_o_) of the control (annealed without GSE) decreased at a longer annealing time within each annealing temperature (Table 4). The GSE-treated chickpea starch exhibited a reduction in m_o_ at 0.1 mL GSE and an increase after 1.0 mL GSE as well as a longer annealing time, regardless of annealing temperature. The same trend was observed for the C and K parameters. In particular, the downward trend of m_o_ with respect to increasing annealing time reflects a reduction in hygroscopicity, which goes along with longer annealing time and temperature. This may be attributed to a reduction in the total sorption capacity of the material, which may in turn reflect annealing.

**Table 4:** GAB parameters for moisture sorption isotherms of chickpea starch pre-gelatinization/germinated sorghum extract annealed at 40, 50, and 60 °C at 30 and 60 min (m_o_ g/100 g water dry basis).

40 °C						
		m_o_	Cg	K	R^2^	RMSE
30 min	No GSE	0.14 ± 0.01 ^b^	2.47 ± 0.02 ^b^	0.41 ± 0.03 ^b^	0.99	0.213
	0.1 mL	0.18 ± 0.01 ^a^	2.51 ± 0.03 ^b^	0.45 ± 0.03 ^b^	0.99	0.740
	1.0 mL	0.18 ± 0.02 ^a^	3.20 ± 0.04 ^a^	0.51 ± 0.02 ^a^	0.99	0.860
60 min	No GSE	0.12 ± 0.03 ^c^	2.05 ± 0.04 ^c^	0.25 ± 0.01 ^c^	0.99	0.871
	0.1 mL	0.17 ± 0.02 ^b^	2.90 ± 0.04 ^b^	0.46 ± 0.02 ^b^	0.99	0.923
	1.0 mL	0.38 ± 0.04 ^a^	3.91 ± 0.06 ^a^	0.53 ± 0.04 ^a^	0.99	0.914
**50 °C**						
30 min	No GSE	0.14 ± 0.02 ^b^	2.75 ± 0.08 ^c^	0.36 ± 0.07 ^b^	0.99	0.741
	0.1 mL	0.16 ± 0.02 ^b^	2.96 ± 0.18 ^b^	0.46 ± 0.05 ^a^	0.99	0.768
	1.0 mL	0.21 ± 0.01 ^a^	3.74 ± 0.12 ^a^	0.48 ± 0.04 ^a^	0.99	0.613
60 min	No GSE	0.12 ± 0.02 ^c^	2.46 ± 0.09 ^b^	0.29 ± 0.03 ^b^	0.99	0.417
	0.1 mL	0.16 ± 0.02 ^b^	2.65 ± 0.15 ^b^	0.51 ± 0.03 ^a^	0.99	0.560
	1.0 mL	0.29 ± 0.02 ^a^	3.62 ± 0.15 ^a^	0.56 ± 0.03 ^a^	0.99	0.951
**60 °C**						
30 min	No GSE	0.14 ± 0.04 ^b^	2.08 ± 0.13 ^b^	0.29 ± 0.04 ^b^	0.99	0.921
	0.1 mL	0.27 ± 0.03 ^a^	2.26 ± 0.17 ^b^	0.33 ± 0.03 ^b^	0.99	0.731
	1.0 mL	0.32 ± 0.04 ^a^	2.85 ± 0.15 ^a^	0.51 ± 0.05 ^a^	0.99	0.860
60 min	No GSE	0.11 ± 0.03 ^b^	1.72 ± 0.08 ^b^	0.24 ± 0.01 ^b^	0.99	1.035
	0.1 mL	0.44 ± 0.05 ^a^	1.81 ± 0.10 ^b^	0.24 ± 0.04 ^b^	0.99	0.994
	1.0 mL	0.44 ± 0.02 ^a^	3.23 ± 0.14 ^a^	0.58 ± 0.02 ^a^	0.99	1.004

m_o_ = monolayer moisture content; RMSE = root-mean-square error; C and K are GAB parameters related to monolayer and multilayer properties. Values (means ± S.D) followed by different letters under particular annealing temperature and time within each column are significantly different.

Induced physical structural changes on the starch granules during annealing and before gelatinization. These changes appeared to influence the way starch granules go through the gelatinization events, such as granule swelling rate, which has a direct effect on the molecular weight profile of the gelatinized starch. Keeping in mind, the granule structure and the entanglement between amylose and amylopectin as well as their ratio have a direct effect on the events leading to starch gelatinization. The 0.1 mL GSE treatment had little effect on the m_o_ within each annealing temperature, but the 1.0 mL GSE increased the m_o_ values indicating an increase in the hygroscopicity of the PGS. This could be ascribed to the exposure of additional hydroxyl groups which allowed for more water binding sites, due to the action of α–amylase. The comparison of annealing chickpea starch at different temperatures within 30 or 60 min is presented in Table 4. The data showed very little difference between m_o_ values for the chickpea starch control after annealing for 30 min regardless of annealing temperature, whereas annealing for 60 min at 60 °C showed higher m_o_. Samples treated with GSE at 50 °C exhibited lower m_o_ after both annealing times, which indicates the effect of annealing on the starch granules structure that affected the gelatinization events leading to different molecular profiles of the PGS. Therefore, the gelatinization product which is the substrate for α-amylase will produce different molecular sizes leading to different hygroscopicity. It is also apparent how chickpea starch treated with 1.0 mL GSE and annealed at 60 °C, exhibited the highest m_o_ compared to the control. Therefore, GSE treatment has a direct effect on the PGS. The m_o_ of the sweet potato starch was dependent on annealing temperature and GSE treatment, as well. Compared to chickpea starch, the native S.P.S had higher m_o_ at higher annealing temperatures and time (Table 5). Higher hygroscopicity was recorded for 0.1 mL GSE at higher temperatures and a short time, but m_o_ exhibited a drop after 1.0 mL GSE treatment. Once again, annealing in 0.1 mL GSE for 30 and 60 min, the m_o_ increased at higher temperature indicating a more hygroscopic material, but 1.0 mL GSE produced a less hygroscopic material due to the low m_o_. The difference between the m_o_ of chickpea starch and sweet potato starch could be attributed to the different amylose content which has a direct effect on the granule structure of the native starch. The activity of α–amylase appeared to be dependent on the molecular structure of the gelatinized starch. An example of PGS isotherm profiles showing the presence of hysteresis between adsorption and desorption profiles is presented in Figure 1. The hysteresis effect extended over the entire water activity range for both starches but it was most pronounced in the 0.6 < a_w_ < 0.8 region. The magnitude of the hysteresis loop is larger for the samples annealed at 50 °C compared to the other temperatures. It is obvious how annealing temperature can affect the adsorption and desorption as well as the hysteresis, which indicates the ability of the PGS to lose water in a different pathway than up-taking. It is also apparent the effect of GSE treatment on the profiles is due to the action of α-amylase. Therefore, PGS absorb moisture faster, but the desorption profile indicates a stronger association between the water and the PGS which is obvious in Figure 1, where at the same water activity, the desorption profile exhibited moisture content higher than adsorption [12].

**Table 5:** GAB parameters for moisture sorption isotherms sweet potato starch pre-gelatinization/germinated sorghum extract annealed at 40, 50, and 60 °C at 30 and 60 min (m_o_ g/100 g water dry basis).

40 °C						
		m_o_	Cg	K	R^2^	RMSE
30 min	No GSE	0.18 ± 0.02 ^a^	3.20 ± 0.11 ^b^	0.41 ± 0.02 ^b^	0.99	0.864
	0.1 mL	0.18 ± 0.01 ^a^	2.46 ± 0.17 ^c^	0.45 ± 0.01 ^a^	0.99	0.208
	1.0 mL	0.14 ± 0.03 ^a^	3.54 ± 0.12 ^a^	0.49 ± 0.05 ^a^	0.99	0.743
60 min	No GSE	0.12 ± 0.02 ^b^	3.90 ± 0.09 ^a^	0.53 ± 0.04 ^a^	0.99	0.603
	0.1 mL	0.17 ± 0.03 ^b^	2.89 ± 0.12 ^b^	0.46 ± 0.03 ^a^	0.99	0.572
	1.0 mL	0.38 ± 0.02 ^a^	2.05 ± 0.10 ^c^	0.25 ± 0.02 ^b^	0.99	0.518
**50 °C**						
30 min	No GSE	0.16 ± 0.03 ^b^	2.96 ± 0.10 ^b^	0.47 ± 0.04 ^a^	0.99	0.206
	0.1 mL	0.21 ± 0.02 ^a^	2.75 ± 0.17 ^b^	0.36 ± 0.03 ^b^	0.99	0.385
	1.0 mL	0.14 ± 0.04 ^b^	3.74 ± 0.14 ^a^	0.47 ± 0.02 ^a^	0.99	0.742
60 min	No GSE	0.29 ± 0.02 ^a^	2.46 ± 0.12 ^b^	0.28 ± 0.01 ^b^	0.99	0.552
	0.1 mL	0.16 ± 0.03 ^b^	2.64 ± 0.09 ^b^	0.50 ± 0.04 ^a^	0.99	0.044
	1.0 mL	0.12 ± 0.03 ^b^	3.62 ± 0.12 ^a^	0.56 ± 0.05 ^a^	0.99	0.554
**60 °C**						
30 min	No GSE	0.27 ± 0.03 ^a^	2.26 ± 0.11 ^b^	0.33 ± 0.04 ^b^	0.99	0.920
	0.1 mL	0.32 ± 0.04 ^a^	2.08 ± 0.20 ^b^	0.29 ± 0.04 ^b^	0.99	0.734
	1.0 mL	0.13 ± 0.03 ^b^	2.85 ± 0.11 ^a^	0.51 ± 0.05 ^a^	0.99	0.960
60 min	No GSE	0.44 ± 0.03 ^a^	1.80 ± 0.09 ^b^	0.24 ± 0.02 ^b^	0.99	0.687
	0.1 mL	0.46 ± 0.04 ^a^	1.72 ± 0.10 ^b^	0.24 ± 0.02 ^b^	0.99	0.908
	1.0 mL	0.11 ± 0.04 ^b^	3.22 ± 0.12 ^a^	0.57 ± 0.01 ^a^	0.99	0.232

m_o_ = monolayer moisture content; RMSE = root-mean-square error; C and K are GAB parameters related to monolayer and multilayer properties. Values (means ± S.D) followed by different letters under particular annealing temperature and time within each column are significantly different.

## 3. Materials and Methods

### 3.1. Starch Isolation

The starches used in this study were obtained from several sources. Chickpea (*Cicer arietinum* L.) starch (C.P.S), white bean (*Phaseolus vulgaris* L.) starch (W.B.S), sweet potato (*Ipomoea batatas* L.) starch (S.P.S), and wheat (*Triticum aestivum* L.) starch (W.S) were extracted from raw materials acquired from a local market in Riyadh, Saudi Arabia. Corn (*Zea mays* L.) starch (C.S) was generously supplied by ARASCO Company, also located in Riyadh, Saudi Arabia. The authors have already provided an in-depth description of the techniques employed for starch isolation in a prior paper [19]. The *Aspergillus* fungal α-amylase (EC3.2.1.1) and sulfuric acid were procured from Sigma Aldrich, a reputable supplier based in St. Louis, MI, USA. The centrifugation process for starch isolation was conducted using a Beckman Centrifuge (Beckman JXN, Brea, CA, USA).

### 3.2. Starch Modification

Sorghum (*Sorghum bicolor* L. Moench) seeds were germinated for four days at 24 °C and 25% moisture, air dried, and 10 g were added to 40 mL distilled water, agitated for 15 min, filtered through Whitman 40, and centrifuged for 10.0 min at 2000× *g*. Germinated sorghum extract (GSE) was given to the supernatant. Starch to water slurry 1:9 (*w*/*v*) (30 g + 270 mL water) and 1.0 mL or 10.0 mL GSE was added to the starch slurry, with starch slurry without GSE serving as the control. The slurry was agitated and annealed in water baths at 40, 50, and 60 °C for 30 or 60 min before being centrifuged three times with fresh water to remove the excess enzyme. After washing, the starch was air-dried with 100 mL of acetone. The dried starch was sieved through a 250 μm sieve and kept at −20 °C for later investigation. The α -amylase activity in the GSE extract was calculated by measuring the activity of a known concentration of pure amylase enzyme solution.

### 3.3. Pre-Gelatinized Starch

Native or GSE-treated starch slurries were prepared by adding 70 g starch (14% MC) to 200 mL distilled water. A thin layer of the slurry was spread on a heated flatbed until a dry thin layer (sheet) was formed (about 0.8 mm). The sheet was left to take room temperature, ground in a coffee grinder to pass through a 250 μm sieve and sealed in plastic bags, labeled as pre-gelatinized starches (PGS), and stored in the refrigerator for further analysis.

### 3.4. Water Holding Capacity

Beuchat [30] method was used to calculate the water holding capacity (WHC). In 5 mL of distilled water, 0.1 g of PGS (W0) was suspended and vortexed for 10 s. After 30 min at ambient temperature (25 ± 2 °C), the sample was centrifuged at 2000× *g* for 15 min, and the precipitate was weighed (W1). According to the following relationship, the WBC was determined as grams of water absorbed per gram of starch: WHC (g/g) = W1 − W0/W0.

### 3.5. Sugars Determination

A 10% glucose standard solution (0.2 mg glucose/2 mL) was prepared and a standard curve was constructed using 0, 50, 100, and 150 µg glucose/mL and the absorption was read at 490 nm. The phenol–sulfuric acid method as described by [31] was used for the determination of sugars with some modification. Pre-gelatinized starch (0.1 g) was added to 10 mL of double distilled water, vortexed for 60 s, and placed for 60 min at room temperature with occasional hand mixing. The sample was centrifuged for 10.0 min at 2000× *g* and 200 µL aliquot was transferred and diluted with double distilled water to a total of 2 mL. The diluted sample was mixed with 50 µL of 80% aqueous phenol solution and subsequently with 5 mL of concentrated sulfuric acid (98% by weight) and mixed. After 10 min, the sample was vortexed for 30 s and placed for 20 min in a water bath at room temperature for color development. Then the absorption was read at 490 nm.

### 3.6. Freezable Water

Freezable water (FW)of PGS was determined for starches annealed at 40 °C in 1.0 mL GSE for 60 min. The freezable water was determined using differential scanning calorimeter (DSC) analysis by scanning the samples at 10 °C/min using TA instrument DSC (TA instrument, New Castel, PA, USA). PGS sample (10–12 mg at 8% moisture content) was placed in aluminum pans and 18–20 µL distilled water was added, whereas the reference pan contained a similar weight of distilled water. After sealing, the sample was equilibrated for 2 h and cooled from 25 °C to −80 °C, equilibrated for 10 min, and heated to 50 °C. Onset and peak temperature and ΔH were determined using the software provided by TA instruments (Q2000, TA Instruments Inc., New Castle, DE, USA). The DSC was calibrated for temperature and heat flow using indium (melting point: 156.6 °C, ΔH = 28.47 J/g) and pure water (melting point: 0 °C, ΔH = 334.10 J/g). The freezable water was calculated by dividing the ΔH of the melted ice in the sample by the ΔH of the melting ice of pure water.

### 3.7. Sorption Isotherms Determination

PGS moisture sorption isotherms were determined gravimetrically using a sorption analyzer Q5000 SA (TA instruments, New Castile, DE, USA). The sample (10 mg) was loaded on the Q5000 autosampler using quartz pans and the relative humidity was automatically set between 10 and 90% (0.1–0.9 a_w_) at 25 °C. The instrument provided the equilibrium moisture content (EMC) directly after each step. The EMC at the specific water activity is used for the determination of the GAB and BET parameters.

### 3.8. Sorption Isotherm Models

In order to determine the best fit corresponding to a_w_ at the selected temperature, GAB and BET models were applied. The GAB model is represented by the following relationship:

$$\mathrm{Wm}=\frac{C.K.a_{w}.{\;m}_{o}}{\left(1-k.a_{w}\right).\left(1-k.a_{w}+C.\;K\;.{\;a}_{w}\right)}$$

where a_w_ is the water activity, Wm is the equilibrium moisture content and m_o_ is the monolayer moisture content. C and K are GAB constants (C is a constant related to the heat of sorption of the first layer. K is related to the heat of adsorption of the multi-layer) derived from the following polynomial equation:

$$\frac{a_{w}}{\mathrm{EMC}}=\;\alpha {\left({\;a}_{w}\right)}^{2}+\;\beta +\gamma$$

where a_w_ is the water activity and EMC is the equilibrium moisture content. The terms α, β, and γ can be calculated by non-linear regression of the experimental EMC as a function of a_w_. Therefore, the C and K values can be obtained as follows.

$$\alpha =\frac{k}{m_{o}}\left[\frac{1}{C}-1\right]$$

$$\beta =\frac{1}{m_{o}}\left[1-\frac{2}{C}\right]$$

$$\gamma =\frac{1}{m_{o}{}_{.}C.K}$$

$$C=\frac{T+\sqrt{T^{2}-4T}}{2}$$

$$T=\frac{\beta^{2}}{-\alpha \gamma }+4$$

$$K=\frac{1}{C.m_{o}.\gamma }$$

$$m_{o}=\left[1-\frac{2}{C}\right]\times \frac{1}{\beta }$$

Therefore, EMC can be estimated by rearranging following equation

$$\frac{m_{o}}{\mathrm{EMC}}=\frac{\left[1-a_{w}\right]\left[1+\left(C-1\right)\right]a_{w}}{C.{\mathrm{Ka}}_{w}}$$

where m_o_ is the monolayer moisture content and EMC is the equilibrium moisture content.

The BET model is represented by

$$m=\frac{{C\;a}_{w}m_{o}}{\left(1-a_{w}\right)\left[1+\left(C-1\right)\right]a_{w}}$$

After arrangement, the BET equation is as follows:

$$\frac{m_{o}}{\mathrm{EMC}}=\frac{\left[1-a_{w}\right]\left[1+\left(c-1\right)\right]}{C.a_{w}}$$

### 3.9. Model Validation

The GAB and BET models are fitted to a non-linear regression equation. All calculations were made using Sigma Plot version 10.0. Besides the R^2^ of the non-linear regression, the goodness of the model fit was tested using the percentage root square error (RMSE), which is the absolute measure of the fit of the model [29].

$$\;\;\;\mathrm{RMSE}=\sqrt{\frac{\sum_{i=1}^{N}(m^{i}{}_{e\;}-m^{i}{}_{p)}}{N}}$$

where m_e_ is the experimental EMC value and m_p_ is the predicted value and N is the number of experimental data.

### 3.10. Statistical Evaluation

All the observations were recorded in triplicate. ANOVA was used to examine the experimental data, which were expressed as mean and standard deviation. Duncan’s multiple range test was used to determine whether there were significant differences between experimental mean values (*p* ≤ 0.05). SAS Foundation 9.2 for Windows (SAS Institute, Inc., Cary, NC, USA) was used to analyze the data.

## 4. Conclusions

Sweet potato starch exhibited the highest water holding capacity and the lowest freezable water, whereas chickpea starch had the most freezable water. Therefore, it is probable to state that much more stable products may be obtained if sweet potato starch is present in starch-rich foods. The moisture sorption of different pre-gelatinized starches increased with increasing water activity at different annealing temperatures and was characterized as Type II isotherm. The equilibrium moisture content and monolayer moisture contents (m_o_) decreased after treatment with 0.1 mL GSE but increased after 1.0 mL GSE-treatment and longer annealing time. The results showed that α-amylase had a significant effect on the equilibrium moisture content and monolayer moisture content (m_o_) of the starches. The GAB model showed a high correlation coefficient of determination (R^2^) and a low percentage square root error (RMSE), indicating the best fit of the experimental data in the whole range of water activity. These fundamental data are important in assessing the applicability of starch in food and pharmaceutical industries.

## Figures and Tables

**Table 1:** The water holding capacity of pre-gelatinized, native, and modified starches.

		Chickpea Starch	Corn Starch	White Bean Starch	Wheat Starch	Sweet Potato Starch
	Native	10.12 ± 1.48 ^b^	4.51 ± 0.89 ^e^	6.80 ± 0.4 ^c^	5.93 ± 1.14 ^d^	13.00 ± 0.64 ^a^
40 °C						
30 min	No GSE	11.43 ± 0.15 ^b^	5.40 ± 0.12 ^d^	7.84 ± 0.19 ^c^	5.28 ± 0.30 ^d^	13.47 ± 0.11 ^a^
	0.1 mL	15.09 ± 0.18 ^a^	5.28 ± 0.13 ^e^	11.10 ± 0.16 ^c^	6.19 ± 0.24 ^d^	13.90 ± 0.12 ^b^
	1.0 mL	17.76 ± 0.23 ^a^	5.19 ± 0.01 ^e^	13.73 ± 0.20 ^c^	6.86 ± 0.10 ^d^	14.32 ± 0.15 ^b^
60 min	No GSE	11.29 ± 0.10 ^b^	8.39 ± 0.13 ^c^	8.65 ± 0.21 ^c^	6.54 ± 0.41 ^d^	16.15 ± 0.21 ^a^
	0.1 mL	14.60 ± 0.13 ^b^	9.90 ± 0.16 ^d^	12.62 ± 0.24 ^c^	6.55 ± 0.14 ^e^	16.82 ± 0.16 ^a^
	1.0 mL	17.00 ± 0.32 ^ab^	9.40 ± 0.26 ^e^	13.34 ± 0.31 ^c^	10.61 ± 0.10 ^d^	17.81 ± 0.21 ^a^
50 °C						
30 min	No GSE	12.04 ± 0.60 ^b^	6.08 ± 0.20 ^e^	9.35 ± 0.30 ^c^	7.83 ± 0.2 ^d^	14.35 ± 0.32 ^a^
	0.1 mL	14.61 ± 0.10 ^b^	6.74 ± 0.10 ^e^	10.52 ± 0.10 ^c^	9.54 ± 0.09 ^d^	16.73 ± 0.23 ^a^
	1.0 mL	16.51 ± 0.60 ^b^	7.54 ± 0.10 ^e^	13.03 ± 0.10 ^c^	9.78 ± 0.15 ^d^	17.01 ± 0.14 ^a^
60 min	No GSE	12.06 ± 0.20 ^b^	8.22 ± 0.30 ^d^	9.95 ± 0.10 ^c^	8.40 ± 0.16 ^d^	15.60 ± 0.18 ^a^
	0.1 mL	14.63 ± 0.50 ^b^	9.03 ± 0.30 ^d^	10.88 ± 0.20 ^c^	8.37 ± 0.21 ^e^	17.11 ± 0.13 ^a^
	1.0 mL	13.65 ± 0.10 ^c^	9.28 ± 0.10 ^e^	14.61 ± 0.10 ^b^	10.46 ± 0.26 ^d^	18.22 ± 0.21 ^a^
60 °C						
30 min	No GSE	7.18 ± 0.24 ^c^	5.56 ± 0.53 ^d^	8.38 ± 0.26 ^b^	Gelatinized	10.39 ± 0.14 ^a^
	0.1 mL	7.84 ± 0.12 ^c^	5.47 ± 0.32 ^d^	10.40 ± 0.61 ^b^	Gelatinized	11.82 ± 0.11 ^a^
	1.0 mL	8.95 ± 0.23 ^c^	5.43 ± 0.41 ^d^	10.53 ± 0.36 ^b^	Gelatinized	12.00 ± 0.12 ^a^
60 min	No GSE	8.18 ± 0.23 ^c^	8.13 ± 0.12 ^c^	9.22 ± 0.36 ^b^	Gelatinized	14.93 ± 0.12 ^a^
	0.1 mL	8.29 ± 0.23 ^c^	8.80 ± 0.12 ^c^	10.80 ± 0.12 ^b^	Gelatinized	15.26 ± 0.23 ^a^
	1.0 mL	9.29 ± 0.51 ^c^	9.80 ± 0.23 ^c^	11.17 ± 0.12 ^b^	Gelatinized	15.42 ± 0.28 ^a^

Values (means ± S.D) followed by different letters within each row are significantly different.

**Table 2:** Soluble sugars of pre-gelatinized native and annealed with and without GSE.

	Glucose (µg/mL)		
Starch Type	Native	Annealed	Annealed with GSE
White bean	24.93 ± 0.56 ^d^	14.93 ± 0.08 ^d^	82 ± 2.01 ^d^
Chickpea	33.16 ± 1.11 ^c^	33.10 ± 1.02 ^b^	104 ± 1.21 ^b^
Corn	20.94 ± 0.98 ^e^	17.21 ± 1.33 ^c^	92 ± 2.54 ^c^
Wheat	39.49 ± 1.32 ^b^	35.18 ± 3.21 ^b^	116 ± 3.34 ^a^
Sweet potato	96.13 ± 2.04 ^a^	77.02 ± 2.27 ^a^	79 ± 2.59 ^d^

Values (means ± S.D) followed by different letters within each column are significantly different.

**Table 3:** Freezable water.

Starch Type	Onset (°C)	Peak (°C)	ΔH (J/g)	Freezable Water
White bean	−3.99 ± 0.01 ^d^	9.34 ± 0.21 ^a^	514.2 ± 12.32 ^b^	1.52 ± 0.02 ^b^
Chickpea	−4.56 ± 0.02 ^c^	5.12 ± 0.10 ^c^	536.3 ± 4.56 ^a^	1.59 ± 0.01 ^a^
Corn	−4.98 ± 0.10 ^b^	6.08 ± 1.02 ^b^	286.1 ± 8.21 ^e^	0.85 ± 0.02 ^d^
Sweet potato	−8.44 ± 0.12 ^a^	3.77 ± 0.21 ^d^	204.3 ± 11.20 ^f^	0.60 ± 0.01 ^e^
Wheat	−8.15 ± 0.13 ^a^	−0.17 ± 0.01 ^f^	402.6 ± 3.25 ^c^	1.19 ± 0.03 ^c^
Pure water	2.15 ± 0.09 ^d^	1.34 ± 0.05 ^e^	337.4 ± 4.87 ^d^	-

Values (means ± S.D) followed by different letters within each column are significantly different.

## Data Availability

Not applicable.
